# Intraoperative computed tomography for detection of residual stones in endourology procedures: systematic review and meta-analysis

**DOI:** 10.1590/S1677-5538.IBJU.2024.0092

**Published:** 2024-04-07

**Authors:** Henrique L. Lepine, Fabio C. Vicentini, Eduardo Mazzucchi, Wilson R. Molina, Giovanni S. Marchini, Fabio C. Torricelli, Carlos A. Batagello, Alexandre Danilovic, William C. Nahas

**Affiliations:** 1 Faculdade de Medicina da Universidade de São Paulo São Paulo SP Brasil Faculdade de Medicina da Universidade de São Paulo, São Paulo, SP, Brasil;; 2 Universidade de São Paulo Faculdade de Medicina Departamento de Urologia do Hospital das Clínicas São Paulo SP Brasil Departamento de Urologia do Hospital das Clínicas da Faculdade de Medicina da Universidade de São Paulo, São Paulo, SP, Brasil;; 3 University of Kansas Endourology Section Department of Urology Kansas KS USA Department of Urology, Endourology Section, University of Kansas, Kansas City, KS, USA

**Keywords:** Nephrolithotomy, Percutaneous, Ureteroscopy, Meta-Analysis as Topic

## Abstract

**Background:**

Success rates in endourological procedures, notably percutaneous nephrolithotomy (PCNL) and ureteroscopy (URS), have demonstrated suboptimal outcomes, leading to more reinterventions and radiation exposure. Recently, the use of intraoperative computed tomography (ICT) scans has been hypothesized as a promising solution for improving outcomes in endourology procedures. With this considered, we conducted a comprehensive systematic review and meta-analysis encompassing all available studies that evaluate the impact of the use of intraoperative CT scans on surgical outcomes compared to conventional fluoroscopic-guided procedures.

**Methods:**

This systematic review was conducted in accordance with PRISMA guidelines. Multiple databases were systematically searched up to December of 2023. This study aimed to directly compare the use of an ICT scan with the standard non-ICT-guided procedure. The primary endpoint of interest was success rate, and the secondary endpoints were complications and reintervention rates, while radiation exposure was also evaluated. Data extraction and quality assessment were performed following Cochrane recommendations. Data was presented as an Odds ratio with 95%CI across trials and a random-effects model was selected for pooling of data.

**Results:**

A comprehensive search yielded 533 studies, resulting in the selection of 3 cohorts including 327 patients (103 ICT vs 224 in non-ICT). Primary outcome was significantly higher in the experimental group versus the control group (84.5% vs 41.4% respectively, 307 patients; 95% CI [3.61, 12.72]; p<0.00001; I2=0). Reintervention rates also decreased from 32.6% in the control to 12.6% in the ICT group (OR 0.34; 95%CI [0.12,0.94]; p =0.04; I2= 48%), whereas complication rates did not exhibit significant differences. Radiation exposure was also significantly reduced in two of the included studies.

**Conclusion:**

This meta-analysis highlights a favorable outcome with intraoperative CT scan use in PCNL procedures, showing a considerable increase in SFR when compared to standard fluoroscopy and nephroscopy. Despite limited studies, our synthesis underscores the potential of ICT scans to significantly reduce residual stones and their consequences for endourology patients, as reinterventions and follow-up ionizing radiation studies.

## INTRODUCTION

Endourology procedures such as percutaneous nephrolithotomy (PCNL) are commonly performed when dealing with kidney stones larger than 2cm (1). Alternately, retrograde ureteroscopy (URS) using high-power (100W-120W) holmium lasers has also been increasingly performed by urologists (2). In both procedures, the intraoperative use of fluoroscopy has been the standard method for detecting potential residual stones and treating them, even though stone-free rates have shown to be suboptimal (3). Moreover, the surgeon's intraoperative assessment of the stone status is often different from the post-operative CT scan imaging results. In a recent series, Hartung et al. reported a sensitivity of only 24% (57 out of 237) in the capability of the surgeon to predict which patient has residual fragments, compared to a post-operative CT scan (4).

Residual stones are usually detected post-operatively on imaging studies or if the patient returns symptomatic, requiring reinterventions. In both scenarios, morbidity and radiation exposure increase in patients undergoing endourology procedures, with the latter sometimes exceeding occupational exposure limits imposed by the International Commission on Radiological Protection (ICRP) (5, 6). Complications are also intrinsically related to reintervention rates, as well as secondary calyceal stones and duration of intervention, which might be impacted by the use of an ICT (7–9).

Striving to increase visibility and detection of residual stones and possibly decrease radiation exposure in endourology patients, the use of hybrid room imaging has been recently considered as a potential solution. Initial experiences of new imaging technologies in the endourology field were performed in 2006 utilizing high magnification rotational fluoroscopy and later in 2017, when promising initial results using the Uro Dyna-CT scan intraoperatively during PCNL were published (10, 11). Since then, the viability of using ICT and comparisons to the standard fluoroscopy assessment of residual stones has been increasingly targeted for research (12–15).

The principal idea behind intraoperative CT scans is to increase the detection of residual fragments, allowing them to be treated still during the surgery. This may reduce reintervention rates and potentially medical costs, while also decreasing radiation exposure by obviating the need for post-operative imaging studies. The use of this technology during surgery has only been performed by a select number of reference centers worldwide, and this is the first meta-analysis to evaluate and compile available data. In accordance, we conducted a systematic review and meta-analysis involving all the currently available evidence for the use of intraoperative CT scans instead of the standard fluoroscopy for intraoperative guidance during PCNL. Our aim is to synthesize the current evidence and provide a comprehensive analysis of the success rate, risk of reinterventions, complications, and radiation exposure.

## MATERIALS AND METHODS

### Eligibility criteria

In this paper, we included all clinical studies focused on comparing intraoperative CT scan use in PCNL and the use of fluoroscopy for the detection of residual stones. Exclusion criteria comprised studies with different reported outcomes, studies lacking a control group, studies in languages other than English, along with case reports, letters, reviews, and comments. This study was prospectively registered on PROSPERO under the protocol number: CRD42023486708.

### Outcomes

This study directly compared the use of an ICT scan versus the standard fluoroscopy and nephroscopy-guided procedures, upon the hypothesis of allowing for improved surgical outcomes in endourology patients. This study primarily examined differences in the immediate success rates, at the end of the initial procedure (not considering any reintervention), with confirmation of post-operative imaging or by lack of patient readmission at 30-90 days. Secondary outcomes included rates of reintervention, complications, and radiation exposure.

### Screening

After deduplication, where we used Endnote online™ 20 (Clarivate, Philadelphia, PA) (16), two independent researchers (HL and FV) screened the studies by title and abstract, and disagreements were solved by a third author (WM). Following this process, full-text screening was performed.

### Search strategy and risk of bias assessment

We systematically searched multiple databases (Medline, Scopus, Embase, ScienceDirect, and Cochrane) for trials including the terms: "Intraoperative Computed tomography", "Intraoperative CT scan", "Cone beam computed Tomography", "Portable computer Tomography", "Uro Dyna-CT", "Endoscopic Stone Surgery", "Percutaneous Nephrolithotomy" and "Endourology". All the references from the included studies were also manually searched for any additional study that would fit the inclusion criteria. The data extraction was conducted independently by two different authors (HL and FV), following the inclusion and exclusion criteria. The data was subsequently reviewed by two different authors to ensure exactness. Quality assessment was performed with the risk of bias in non-randomized studies of interventions (ROBINS I) (17). Two independent authors completed the risk of bias assessment (HL and FV). Disagreements were resolved through a consensus after discussing reasons for discrepancy.

## Statistical Analysis

Dichotomous data are presented as odds ratio (OR) and was performed with a 95% confidence interval, with a p-value < 0.05. Heterogeneity was examined with the Cochran Q test and I2 statistics, and P values inferior to 0.005 and I2>30% were considered significant for heterogeneity, denoting the use of random-effect analysis (18). Statistical analysis was entirely conducted in Review Manager version 5.4.1 (Nordic Cochrane Centre, The Cochrane Collaboration, Copenhagen, Denmark) (19).

## RESULTS

### Study selection and characteristics

**FIGURE 1:**
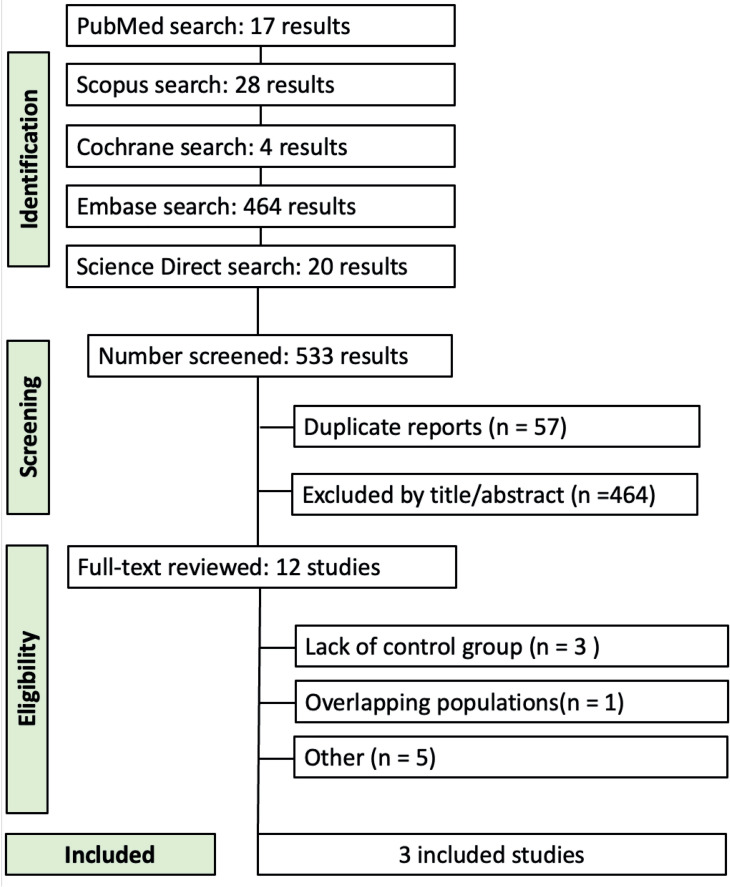
Flow diagram of study screening and selection

A total of 533 results were identified. After the removal of duplicates, 476 articles were screened, and 464 of them were excluded based on title and abstract thorough review. This process resulted in 12 studies, which were selected for full-text revision. Finally, a total of 3 studies met the criteria and were included in the final analysis (Figure-1).

A total of 327 patients were contemplated. All included studies compared a prospective intraoperative CT scan (experimental) against a non-intraoperative CT group during PCNL, collected retrospectively. Since groups were collected at different points in time, and due to the observational nature of the studies, there are some meaningful disparities in baseline characteristics of groups within studies. Van den Broeck et al., for instance, had 75% of patients in the experimental group undergoing mini-PCNL compared to 20% of the control group (p < 0.01), even though both approaches are comparable in terms of effectiveness and complication rates (20). Ultrasonic lithotripters were also less used in the ICT group compared to control (10% vs 45%) (p = 0.01) (12). Regarding Glover et al. populations, supine positioning and tubeless procedures were significantly more used in the ICT group (92% vs 30%, p < 0.001 and 50% vs 7%, p = 0.001, respectively) (14). Cohorts in Patel et al. were similar in baseline characteristics (13). Other baseline characteristics of included studies, including the ICT model used in each study are presented in Table-1 for a comprehensive view.

**Table 1:** Baseline characteristics of included studies.

	Study	Van Der Broeck 2021	Patel 2022	Glover 2023
**Intraoperative CT model**		Artis Zeego cone beam CT-scan (Siemens)	Mobile CT scanner (O-Arm; Medtronic)	Mobile CT scanner (O-Arm; Medtronic) and Artis Zeego cone beam CT-scan (Siemens)
**Number of patients**				
	Intraoperative CT	20	60	23
	Control	20	174	30
	Total	40	234	53
**Female sex**				
	Intraoperative CT	6 (30%)	37 (62%)	2 (8.7%)
	Control	5 (25%)	88 (50%)	3 (10%)
	Total	11 (27.5%)	125 (53%)	5 (9.4%)
**Age**				
	Intraoperative CT	63 (51, 70)*	59 (15) †	72 (52-79) §
	Control	53 (48, 64)*	57 (15) †	66 (49-85) §
	Total	56 (48, 66)*	116	NA
**BMI**				
	Intraoperative CT	26.6 (23.6, 30.5)*	33 (10) †	28.43 (20.39 – 46.75) §
	Control	27 (23.3, 28.4)*	33 (10) †	27.12 (17.48 – 40.50) §
	Total	26.8 (23.6, 30.1)*	NA	NA
**Pre-op size of stones**				
	Intraoperative CT	24.5 (17.8, 33)*	20.4 (9.7) †	3.1 (0.9-11.5) §
	Control	30 (18, 45.8)*	23.5 (13.8) †	3.9 (0.9-10.6) §
	Total	25.5 (18, 37.8)*	NA	NA
**Anticoagulant use**				
	Intraoperative CT	0	NA	6
	Control	1	NA	4
	Total	1	NA	10
**Renal laterality**				
	Left/Right Intraoperative CT	15/5	26/34	7/9
	Left/Right Control	12/8	86/88	6/18
	Total	27/13	174/60	23/30
**Procedure type**				
	ICT (PCNL/Mini-PCNL/URS)	4/15/0	only PCNL	11/1/11
	Control (PCNL/Mini-PCNL/URS)	16/4/0	only PCNL	26/4/0
	Total	20/19/0	only PCNL	37/5/11
**OR/surgery duration**				
	Intraoperative CT	159 (122, 191)*	95 (40) †	210 (90-605) §
	Control	117 (93, 146)*	84 (45) †	235 (130-997) §
	Total	128 (97, 189)*	NA	NA

* Reported in median (Inner Quartile Range) value

† Reported in mean (SD) value

§ Reported in median (RANGE) value

### Pooled analysis of studies

**FIGURE 2:**
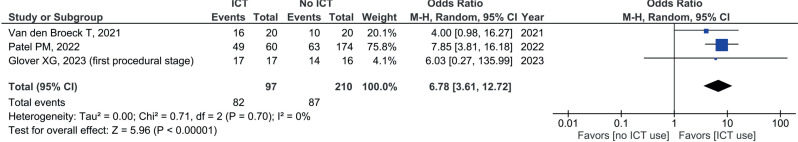
Final success rates.

The primary endpoint, ‘immediate success rate’, exhibited a statistically significant difference between the groups, with no observed heterogeneity. This analysis specifically focused on a subset of patients who underwent a singular procedural stage in Glover et al. This selection was performed to enhance comparability with other included studies. In this context, the success rate in the ICT group was 84.5% (82/97) versus the control group's 41.4% (87/210) across 307 patients. The odds ratio was calculated at 6.78 and depicted in Figure-2 (95% CI [3.61, 12.72]; p < 0.00001; I2 = 0), underscoring a substantial difference in immediate success rates between the two groups.

**FIGURE 3:**
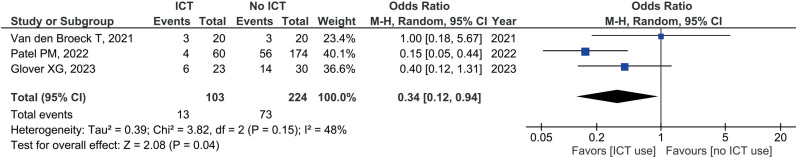
Reintervention rates (at any moment).

**FIGURE 4:**
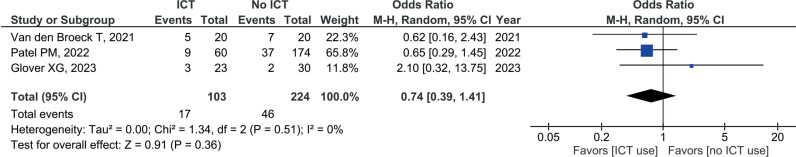
Complication rates.

Reintervention rates at any moment post-operatively were also included as a secondary outcome of our analysis. The comparison showed 13 out of 103 (12.6%) requiring reinterventions compared to 73 out of 224 (32.6%) in the fluoroscopy group, providing a statistically significant difference (Figure-3) (327 patients; OR 0.34; 95% CI [0.12, 0.94]; p = 0.04; I2 = 48%). In contrast, the evaluation of complication rates presented in Figure-4 did not exhibit statistical significance (327 patients; OR 0.74; 95% CI [0.39, 1.41]; p = 0.36; I2 = 0).

Radiation exposure is another crucial point for consideration. Two included studies emphasized effective radiation dose (ERD) measurement and comparison. Patel et al. observed a lower CT-based ERD of 8.4 mSv in the experimental group versus 14.6 mSv in the retrospective cohort (13). Likewise, Glover et al. reported an 83% reduction in longitudinal radiation exposure in the ICT group (15.80 to 2.68 mSv, p < 0.001) (14). Despite favorable results, statistical analysis was not included in this meta-analysis due to a lack of studies that evaluated the extent of radiation exposure.

### Quality assessment of the included studies

**FIGURE 5:**
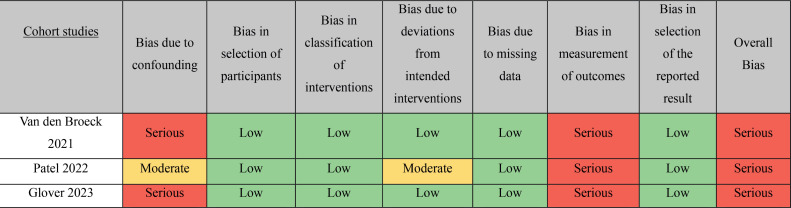
Robins-I tool for risk assessment.

Assessment of the risk of bias in the included studies was performed with the tool for assessing the risk of bias in non-randomized studies of interventions (ROBINS-I) (17) and depicted in Figure-5. All three studies were given a serious risk of bias in measurement of outcomes, given that the criteria for ‘Immediate Success Rate’ wasn't the same in intervention groups. Concerning bias due to confounding, Van den Broeck et al. and Glover et al. also presented serious risk, owing to baseline differences in population characteristics that were not properly adjusted (9, 11). Patel et al. was given moderate risk due to confounding and deviations from intended interventions (10). The overall risk of bias in all studies was deemed serious, on a scale that goes from low to critical risk.

**FIGURE 6:**
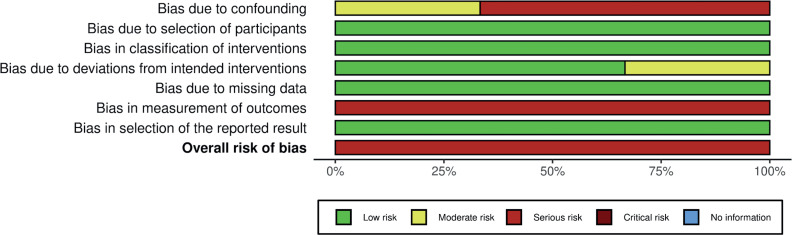
Average risk of bias contribution.

For a better view of the average contribution bias in our study refer to Figure-6.

## DISCUSSION

In this meta-analysis of the three available studies including 327 patients, we have compared the use of intraoperative computed tomography (ICT) against the use of fluoroscopy and nephroscopy for the detection of residual stones in patients undergoing PCNL procedures. Our primary outcome demonstrated that the use of ICT may significantly increase the success rate of the surgery. Reintervention rates and complication rates were also analyzed in this study, with the former showing significant improvement.

**FIGURE 7:**
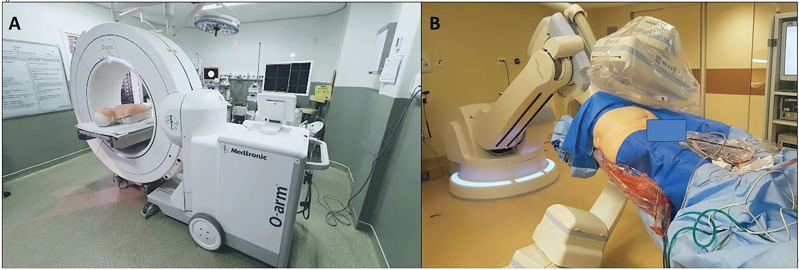
Demonstration of two models of cone beam intraoperative CT scans. 7-A – O-arm® imaging system (Medtronic Inc., Littleton MA), inside an operating room. 7-B – Uro Dyna CT® (Siemens Healthcare Solutions, Erlangen, Germany), showing its use at the end of a PCNL.

Stone-free rate (zero fragments) is regarded as a crucial metric for evaluating the efficacy of interventions in the urolithiasis field (21). Despite recent advances in endourology procedures, stone-free rates are still low (22, 23). The use of CT scans to identify and treat residual stones has been shown to be the most efficacious imaging technique (24), therefore it's hypothesized that limiting them solely to a post-operative setting may be an underuse of its potential. The included studies employed various intraoperative CT imaging devices, showcasing the diverse approaches to enhancing stone-free outcomes. Patel et al. and Van den Broeck et al. utilized cone beam computed tomography machines coupled to O-arm and C-arm, respectively (12, 13). In Glover et al. both types, C and O-arm, were used depending on the availability of the hybrid room (14) (Figure-7). The C-arm technology, initially employed as a high-resolution rotational fluoroscopy system used intraoperatively, demonstrated improved fragment detection and a stone-free rate of 60% in a cohort of 37 patients but wasn't included in the statistical analysis by virtue of no control group (11). Cone-beam CT, on the other hand, can generate 3D imaging based on pyramidal or conical X-ray beams, instead of a fan-shaped beam of a standard CT, providing faster imaging reconstruction (25). While helical CT involves multiple channels and a fan-shaped beam, utilizing high voltage, cone beam imaging, as mentioned earlier, relies on pyramidal or conical X-ray beams, and employs low voltage. This distinction in voltage usage is noteworthy, influencing the efficiency and safety of intraoperative imaging in terms of radiation dose (20).

In our primary analysis, the final success rate was significantly superior in patients undergoing ICT, with 82 of 97 (84.5%) patients considered free of residual stones versus 87 out of 210 (41.4%) in the control group. In this analysis, we have taken into consideration the measurement of success rate in Glover et al. sole including the patients that underwent one procedural stage (first surgery) to account for differences in study designs (14). This considered, even though the use of intraoperative CT scans is possibly a technical challenge and might not be available in all centers, its superiority in terms of detecting residual stones and allowing for prompt removal is noticeable.

Reintervention is a variable greatly associated with residual stones and was also evaluated in this study. The comparison showed a significantly decreased rate of reintervention favoring the ICT group, with 13 out of 103 (12.6%) requiring reintervention compared to 73 out of 224 (32.6%) in the fluoroscopy group. Despite reintervention rates not being directly reported in Glover et al., it was calculated by dividing all patients that needed more than one procedural stage (anesthesia event) by the total number of patients, allowing for comparison to the other included studies (14). This finding supports the evidence provided by the primary outcome and reflects the capability of diminishing morbidity and the elevated economic burden associated with reinterventions in urolithiasis patients (26).

Complications associated with surgery were also included as a secondary outcome for analysis. Even though no statistically significant difference was noted between groups, the use of an intraoperative CT scan is unlikely to increase the rate of complications as long as it does not prolong the surgery excessively. In this regard, studies reported increased surgery duration in the ICT group, but results were not comparable due to dissimilarities in the reported measurements and lack of standard deviation values.

Radiation exposure is another important aspect of endourological procedures, given that patients often undergo multiple imaging studies for the detection of residual stones. Effective radiation dose (ERD) measurement and comparison was a point emphasized in two of the included studies. Patel et al. reported a lower CT-based ERD of 8.4 mSv in the experimental versus 14.6 mSv in the retrospective cohort. Furthermore, Glover et al. reported a decrease in 83% of the longitudinal radiation exposure in the ICT group (15.80 to 2.68 mSv, p < 0.001) (14). These results are explained by the inherently lower doses of the ICT imaging studies compared to the traditional fan-shaped CT scans, as previously discussed. Also, the ICT often relieves the need for a control post-operative CT scan aimed at detecting potential residual stones, further decreasing radiation exposure. This finding holds significant relevance, particularly considering recent efforts within the medical community to systematically diminish radiation exposure from medical devices. This is particularly important in the field of urolithiasis, where patients are routinely subjected to substantial radiation over extended periods (6, 27).

There are some limitations to our study. The most noticeable one is the small number of studies included, relating to the incipient nature of the subject. There are a few other studies in the literature that also evaluated the viability of ICT in PCNL showing positive outcomes, but they were not included by virtue of no control group for comparison (15, 28). In addition, the three included studies in our paper had some structural differences. Among them, the definition of ‘success rate’ and the number of ICTs performed during the same procedure varied across studies, with Glover et al. being distinct from the other ones (14). These differences, however, were taken into consideration in our analysis, as previously reported in this discussion. Concerning heterogeneity, it was not significant for the outcomes included, except for reintervention rates. Another limitation of our study is the lack of cost analysis utilizing ICT compared to standard fluoroscopic PCNL. Unfortunately, none of the publications utilized in this metanalysis addressed this issue in a meaningful way. We acknowledge the high initial acquisitive equipment cost, however, in centers where the CT scanners are already available in intervention radiology suites, the use by urology for stone procedures has the potential to dilute their initial cost due to more procedures being performed.

The use of an ICT scan can provide better visualization of residual stones during PCNL, prompting immediate removal and improving success rates. Moreover, this has a direct positive impact on reintervention rates, which was also evaluated in this study. Less need for additional procedures should be considered for a more robust cost analysis.

The lack of standardized ICT use protocols regarding dose, patient positioning, and surgical table is still an issue. Even though this meta-analysis included a small number of studies, it is the first to approach this topic, one still incipient in the literature and still non-existent in standard clinical practice. Hence, the data synthesized in this meta-analysis serves to display the potential of ICT to improve outcomes and also to instigate new research on the subject.

## CONCLUSION

The results of this meta-analysis suggest that the use of ICT scans during PCNL significantly increases success rates when compared to the standard fluoroscopy-guided detection of residual stones. Our findings also indicate decreased reintervention rates, with no statistically significant differences in complication rates. Despite the paucity of studies included, we have synthesized the currently available evidence in the matter, also presenting its potential to greatly reduce residual stones and their burden in PCNL. This considered, the development of new research on the topic is needed, regarding not only the outcomes evaluated in this study but also other potential benefits of ICT use, such as decreasing total radiation doses.
